# Exploring barriers of childhood full vaccination among children living in Siraro District, West Arsi Zone, Oromia region, Ethiopia: A qualitative study

**DOI:** 10.3389/fped.2023.1083358

**Published:** 2023-03-03

**Authors:** Ararso Hordofa Guye, Tadesse Nigussie, Mengistu Tesema, Dame Banti Shambi, Berhanu Senbeta Diriba, Esayas Mekonen Tefera, Yeabsira Girma

**Affiliations:** ^1^ Department of Public Health, College of Health Sciences, Salale University, Fiche, Ethiopia; ^2^ Department of Pediatrics and Child Health, College of Health Sciences, Salale University, Fiche, Ethiopia; ^3^ Department of Maternal and Child Health, Center for Family Service Organization, Addis Ababa, Ethiopia

**Keywords:** barriers to full vaccination, exploring vaccination barriers, immunization, Siraro District, West Arsi Zone

## Abstract

**Background:**

Childhood immunization is one of the most effective global public health interventions to reduce childhood morbidity and mortality. However, some children remain not fully vaccinated in developing countries due to defaulting from full vaccination, which can put them at risk of acquiring vaccine-preventable disease outbreaks. The barriers to full vaccination were well explored in Ethiopia using a qualitative approach. The study aimed to explore barriers to full childhood vaccination in Siraro District, West Arsi Zone, Oromia, Ethiopia.

**Methods:**

A qualitative study was conducted in Siraro District through 15 key informant health workers interviews and 6 in-depth interviews with children’s mothers from April 20 to May 15, 2022. Data were collected by semi-structured questionnaires and captured using audio tape recorders and field note-taking. A heterogeneous purposive sampling technique was used to select representative study participants. Data transcription and translation were done according to the respondents’ verbatim from the local language to English. Data coding and key categories were identified and analyzed using thematic analysis. Finally, data were presented in narrative forms using respondents' own words as an illustration.

**Result:**

Twenty-one study participants were interviewed and included in this study. Of the explored barriers to full childhood vaccination, the evidence from the respondents was integrated from subcategories and presented as a whole within each thematic area. Five thematic areas emerged from interviews of the participants through thematic analysis of the data. The identified barriers were forgetting the next vaccination schedule, migration of parents, work overload, lack of knowledge and awareness, rumors, and misinformation. Additionally, vaccination service delivery-related barriers such as vaccine vials not being opened for a few children, fear of vaccine side effects, closed health posts during visits by mothers for vaccination, and absence of health extension workers at health posts were the key barriers to full childhood vaccination.

**Conclusion:**

Forgetting vaccination schedule, migration of parents, work overload, rumors, and misinformation, fear of vaccine side effects, vaccine vial not opened for few children, closed health posts during visiting by mothers, absence of health extension workers from health posts were the key barriers to the full vaccination status of children. Thus, the district health office should work on barriers to full vaccination by strengthening vaccination service delivery and improving vaccination awareness through a health extension program.

## Introduction

Childhood vaccination is the process of inducing a protective response in a child's body against a specific disease by administering vaccines and one of the most cost-effective global public health interventions to reduce childhood morbidity and mortality (1, 2). Expanding the universal immunization program is predicted to protect 2.5 million child deaths per year from vaccine-preventable illnesses throughout the world (3). Defaulting from full vaccination is a challenging problem and condition in which a child misses routine vaccination and does not become fully vaccinated for any reason that can put the child at a greater risk of getting vaccine-preventable diseases (VPDs) (3, 4).

Globally, vaccine-preventable diseases are still the most common causes of childhood mortality, with an estimated 2–3 million deaths every year, primarily in Africa and Asia (5). The Partnership for Epidemic Preparedness Innovations, commonly known as the Vaccine Alliance, is a program that promotes the development of potential child vaccines to ensure an adequate level of vaccination and negotiate for their valuing to ensure that low- and middle-income countries have an opportunity to receive all vaccines (6, 7). Immunization has brought comprehensive health to many children globally and has been concrete in protecting children against VPDs in low-and middle-income countries (8, 9). However, vaccination coverage is still a major public health challenge in many third-world countries, particularly in resource-limited areas (10, 11). Even though Africa has achieved remarkable progress in immunization services, large numbers of children remain unvaccinated and undervaccinated (5, 12).

Sub-Saharan African countries still have the highest child mortality rate in the world, and the region had an average child mortality rate of 76 deaths per 1,000 live births in 2019 (10). The occurrence of vaccine-preventable diseases is associated with poor immunization coverage and challenges, and the set up is not fully equipped in sub-Saharan African countries (13). In most of these countries, poor functioning health service delivery systems prevent the efforts to meet immunization targets, especially for children living in hard-to-reach areas. Most die because they cannot access effective interventions tocombat common and preventable childhood illnesses (14, 15). A study conducted in Somalia on factors contributing to childhood immunization uptake revealed that the factors for not fully vaccinating children from health system perspectives were awareness, negative attitude, perceived knowledge of health workers, and missed opportunities. The finding also indicated that the factors from the perspective of individuals and communities were low trust in vaccines, misinterpretation of religious beliefs, vaccine refusals, rumors, and misinformation (16).

In Ethiopia, the barriers to full vaccination against vaccine-preventable diseases are still a major and persistent public health challenge (4, 17). Expanding immunization service is one of the Ethiopian child survival strategies targeted to protect an estimated over 2 million annual childbirths from VPDs, but a significant portion of children have not been vaccinated. As a result, children in the first year of life in Ethiopia have the highest rate of acquiring VPDs such as measles, pneumonia, pertussis, and diphtheria, and the reasons for many of these are believed to be due to barriers to full vaccination (17, 18). The full vaccination coverage has been increased from 39% in 2016 to 43% in the 2019 Ethiopian Demographic Health Survey (EDHS) report, and about 19% of children in the 12–23-month age group have not received any vaccination (19). The highest coverage of full vaccination in Addis Ababa (83%) and the lowest coverage in the Afar region are due to different challenges and opportunities, as explained in other studies (20%) (5, 13).

Studies have also reported that maternal education, place of delivery, antenatal care (ANC) visits, mothers' Tetanus Toxoid (TT) immunization status, postnatal care follow-up, sex of the child, place of residence, and exposure to mass media are predictors of poor child immunization status (8, 20–22). However, most of the studies were quantitative and failed to include certain barriers to full vaccination that could be pertinent in the Ethiopian context, such as socioeconomic status, attitude, service satisfaction, vaccination service delivery, and parent-related barriers to child immunization service provision at the grassroot of individuals and family members. In the selected study area, still in the presence of child vaccination service, some children were not fully vaccinated throughout the year, and the reason for this was not well known. In addition, there were frequent VPD outbreaks such as polio and measles, and independent research on barriers to full vaccination was unavailable, especially using the qualitative approach. The study aimed to explore the barriers to full childhood vaccination in Siraro District, West Arsi Zone, Ethiopia.

## Methods and materials

### Study design, period, and setting

A qualitative phenomenological study was designed to explore barriers to full children vaccination based on the experiences and responsibilities of participants. A qualitative method was chosen to explore and understand barriers to full vaccination at family members, health workers, and vaccination service delivery levels. The study was conducted in Siraro District from April 20 to May 15, 2022. The district is found in the West Arsi Zone and is composed of 4 urban and 28 rural kebeles. According to the Ethiopian context, a kebele is the smallest administrative unit below the district. The district is located in the Southwest of Ethiopia, 64 km from the capital city of zone Shashemene and 314 km from the capital city of the country Addis Ababa.

In line with the national immunization strategies, the district provides Expanded Program on Immunization (EPI) services through static and outreach strategies. Thirty-eight outreach sites provide immunization services to communities living more than 5 km from the nearest health facility. The District Health Office is directly involved in providing support to primary healthcare units through conducting supportive supervision and quarterly review meetings.

According to the Siraro District Health Office estimate in 2021/22, the total population in the district is 217,572, with 35,749 under-five children, and the estimated number of children below 1 year is 7,000. The district has 1 primary hospital, 7 health centers, and 27 functional health posts regarding public health facility coverage, and there are 139 health professionals and 54 health extension workers (HEWs) in the district (Source: Siraro District Health Office Population Profiles).

### Study population

The study population was purposively selected health extension workers from health posts, EPI focal persons working at health centers, primary health care unit (PHCU) directors (PHCUD), District Health Office Head, the EPI coordinator working at the district health office, and mothers/caretakers from the community.

### Eligibility criteria

The inclusion criteria are as follows: Health professionals working in Siraro District and mothers/caretakers living in Siraro District for at least 6 months before the date of data collection were included in this study. The exclusion criteria are as follows: Health workers who worked in the district for less than 6 months by considering no more engagement in the work area due to their having less experience with child vaccination and mothers/caretakers unable to be interviewed due to illness and hearing problems were excluded.

### Sample size and sampling procedures

Fifteen key informants' health professionals working in the district and six mothers/caretakers for in-depth interviews (IDIs) were purposively selected. The participants included four HEWs from health posts, three participants working on the immunization program as a focal person at the Health Center, two participants from the district health office (office head and EPI coordinator), two PHCU directors, and four mothers/caretakers from the community. However, the sample size for the qualitative study was determined on the basis of the ideal saturation of participants.

Key informant interviews and in-depth interviews were conducted with purposively selected 15 key informant healthcare providers and 6 mothers/caretakers using prepared interview guide questions with probing and an audio tape recorder. The study participants were selected using a heterogeneous purposive sampling technique from the primary healthcare unit, district health office, and community.

The health professional participants were selected by considering their working experience with the community, knowledge about child immunization, and service-related responsibilities, and mothers/caretakers were selected if they have a child not fully vaccinated. The interviewed participants were health extension workers from health posts, participants from the healthcare provider working on immunization as a focal person at the health center, participants from district health office working as head and EPI coordinator, PHCU directors from health centers and child mothers/caretakers from the community.

### Operational definitions

#### Full vaccination

A child who received all the recommended routine vaccines as per the national immunization schedule for full vaccination by the age of first birth year was considered fully vaccinated (18).

#### Accessibility and availability of vaccination service

It is the provision of vaccination services by health facilities within walking distance to reach the vaccination site in less than 30 minutes with the presence of the service at all times through alternative methods of immunization service provision including outreach, static, and mobile services (17).

#### Caretaker

A caretaker is the most responsible person who provides care to the child other than the mother for different reasons such as the parent's death, adoption of a child, separation from family, and others (8, 18).

### Data collection tools and procedures

Data collection tools were adapted from a previous study conducted in Ethiopia (20). Data were collected using a semi-structured interview guide for in-depth interviews with mothers and key informants. The interview guides were prepared for key informant health workers and mothers/caretakers separately to probe into the child’s immunization status. For an in-depth interview, eleven interview guide questions were used for key informants, whereas eight interview guide questions were for mothers/caretakers.

Data were collected using an audio tape recorder and field note-taking for each participant, and the interview time ranged from 30 min to 90 min based on the redundancy/saturation of the participants' ideas. Six health professionals who held a master's degree and were fluent in local languages conducted in-depth interviews with key informant health professionals and mothers/caretakers (probing into socioeconomic status, missed opportunities, immunization service delivery systems, any challenges or barriers for no full vaccination of the child, and the reasons for defaulting from full vaccination status of the child). The transcription and translation of data were done from an audio tape recorder for each participant word by word according to their verbatim responses.

### Data quality assurance

The interview guide was prepared in English, translated into Afan Oromo (the local language of the area), and retranslated back to English by another translator who is a health professional to ensure the consistency of the tool. Finally, the Afan Oromo interview guide was used for data collection. The content validity of the tool was cross checked by another health professional. The tools were pretested among similar target populations found in another district before using for actual data collection. Data quality was assured by selecting and keeping a quiet area for interviewing and audio tape-recording with selected study participants. The detailed handwritten field notes of each participant were taken at the time of interviews. The participants were encouraged to speak and express their ideas freely and describe their experience regarding barriers to full vaccination. Interviews were held at the homes of mothers and workplaces of the key informants. All recorded audio tapes of the interviews were transcribed into the local language of the participants (Afan Oromo) and later translated to English.

The transcribed data were cross checked and reviewed by other independent health professionals to compare the consistency and accuracy of the data. The transcription and coding of the data were done according to verbatim of the participants using word-by-word textual note writing. The interview guides were evaluated by qualitative experts from Salale University College of Health Science.

### Data processing and analysis

The interviewed data were first transcribed in the Afan Oromo language, in which the interviews were conducted. Next, the data were translated and transcribed into English by senior language experts at our university. Then, a final edition of the code was developed, and the categories and themes were constructed. Qualitative data analysis was started during data collection and then transcribed and translated to English by replaying the recorded audio tape according to the verbatim of respondents. Data were coded, categorized, and analyzed manually by using inductive thematic analysis (by organizing the topics raised at the time of the in-depth interview independently), and the result was presented in narrative forms to describe barriers to full vaccination.

## Result

Twenty-one study participants (15 key informant health workers and 6 mothers/caretakers) were included. Of the participants, 8 women and 13 men were interviewed. The age of the study participants ranged from 25 to 42 years. The findings by subcategories have been integrated within each thematic area and presented as a whole. However, any specific ideas of the participants identified between these subcategories are highlighted in each theme as appropriate. Five thematic areas that emerged from the in-depth interviews were captured and organized as reported in the following.

### Theme: Parent-related factors

The evidence from in-depth interviews indicated that forgetting the next vaccination schedule, migration of parents from one place to other, work overload, insufficient resources, poor understanding, and geographical location of the residence were the identified barriers for defaulting of the child from full vaccination. A 29-year-old EPI focal person reported that: “…*the economic status of the people in our catchment area is low. Some of the child caretakers are far away from the health facility and can afford to bring their children by motorcycle to vaccinate their children, but those who do not have the resources are less likely to take their children to the health facility. Additionally, the mother's understanding of the status and educational background are not similar in all groups. So, this could delay mothers from returning to health facilities for child vaccination*.”

Another 28-year-old mother mentioned that: “*… I did not bring my child to the health facility by keeping vaccination schedule due to some inconvenience in living houses. In addition, I didn't know when to start child vaccination and when to finish the schedule.”*

The evidence from a 31-year-old health extension worker reported that: “*As to my catchment health post challenges related to child vaccinations; some of the child mothers were migrated from rural villages to urban areas. There is also migration of child parents from place to place, region to region; because we are found on the border area, … child families migrate to the South Nations, Nationalities, and Peoples Region due to some of them having other families and farming land there. Then, as they move from place to place, vaccination interruptions are becoming a major cause of child defaulting.”*

Another 29-year-old mother also reported that: “… *due to frequent absence of drinking water in our local area most of a time we spent many hours in search of getting drinking water after walking long distances. Sometimes we stay day and night fetching water by keeping our orders to fetch water due to overcrowding of people at the source of water. …and also there is work overload in house and then I forget the next vaccination schedule and not returned to health post to vaccinate my child*.”

A 26-year-old EPI focal person reported that: “… *rather than child vaccination, some child parents focus on other supports, especially nutritional screening support commodities such as Plumpnet and others nutritional supplies but they don't bring their child when asked to bring their child for vaccinations. This has been a major cause of defaulting of childhood vaccines*.”

Another 38-year-old respondent from the histrict Health office, an EPI coordinator, mentioned that: “*There are conditions in which some child mothers do not complete the full vaccination of their children due to difficulties they have at home because of work overload. In addition to this, the father of the child does not pay much attention to child vaccination but the burden is only on the mother in all directions and that is the main problem in the social aspects of the parents.”*

### Theme: Knowledge and awareness

The finding from the qualitative study also indicated that not having sufficient knowledge was the factor for defaulting from full vaccination. The 27-year-old mother stated that: “*…including me some child parents did not give attention for child vaccination due to lack of sufficient knowledge and not knowing the benefit of child immunization as well.”*

A 31-year-old PHCUD said that: “*we have identified existing problems related to child vaccination through supportive supervision and that the health extension workers are not providing adequate information for child mothers while vaccinating the children but only writing down the date of the appointment on vaccination card. The health extension workers say to come back next month but they do not give enough explanation in detail to the child's mothers. Then, mothers of children forget the appointment date and stayed at their home without vaccinating child, which causes defaulting of children from vaccination.”*

### Theme: Attitude and service satisfaction

Attitude and service satisfaction was the other thematic area identified from in-depth interviews of study participants. A of 32-year-old mother mentioned that: “… some *health workers means of welcoming the mothers during bringing the child for vaccination is not satisfactory as I think. Unless otherwise, the problem for defaulting of my child from full vaccination is my weakness and not giving attention due to work overload.”*

Another 25-year-old mother stated: “… *the issue of health extension workers saying one vial is not opened for few children is not good for me and … giving appointment and absence of health extension workers from health post is as to me not attractive service provision. Not giving attention to child vaccination is also the other problem for incomplete child vaccination.”* In addition, she said that: “… *when my child becomes feel discomfort as a result of receiving vaccines, I also feel discomfort for my child … and I prefer to default my child from receiving next vaccines.”*

A 27-year-old EPI focal person stated that: “*Some child mothers do not take child vaccines by keeping their order of vaccination at the vaccination center due to they want to vaccinate their child immediately and return to their home. … as a result, the child caretakers' interest to vaccinate their children is lost and they don't want to wait until vaccines are given to other parents*.”

### Theme: Rumors and misinformation

The evidence from the ideas of study participants indicated that rumors and misinformation about child vaccination causes defaulting of children from full vaccination. A 31-year-old mother old mentioned that: “ *There is a problem in a community that I had seen by my eyes … some child caretakers say to the others child parents if the child receives vaccine they believe that child will get the illness. Such rumors and misunderstandings in the community can be preventing some mothers from vaccinating their children.* … *and some wrong information in the village can be complicating us and making fear in our mind.”* Another response from a 28-year-old health extension worker reported that: “… *few child mothers have misinformation and misunderstanding on child vaccination which they say God may protect my child from illness therefore not good to receive vaccines. In addition, some of them believe that vaccination may cause physical disability on children.”*

### Theme: Vaccination service delivery

Concerning the child vaccination service delivery thematic area, the evidence from in-depth interviews of the respondents indicated that lack of information, fear of vaccine side effects, vaccine vial not opened for few children, closed health posts during visits by mothers, and absence of health extension workers from health posts were the reasons for defaulting of children from vaccination.

The 25-year-old mother reported that; “… *as to my child defaulting from getting full vaccination,…. one day when I bring my child for vaccination at 9 months the health extension worker said to me the vaccine is not opened only for your child … and she said this vial can only be opened for 10 children. Again one day when I come back to the health post was closed and I haven't seen a health extension worker at the post, then I didn't return for vaccination next time.”*

Other evidence from the 30-year-old mother indicated that: “… *my child was not completed all recommended vaccines due to the Health Extension worker of our kebele was not available at the health post. Because she was gone to attend her education as I was heard from kebele leaders that is the reason for not receiving all vaccines by my child*.” In addition, the 31-year-old mother stated that: “… *I fear the side effects that can cause fever and strange discomfort for a few days after receiving a vaccination, which is a highly stressful condition until the child, recover from discomforts. My child becomes sick from taking vaccinations, I worry about such stressful events and I never go to health post to vaccinate my child again*.”

The finding also indicated that not properly telling the types of vaccine received and not explaining about child vaccination were the factors for defaulting from full vaccination. The 38-year-old district EPI coordinator reported that*:* “… *some mothers complete child vaccinations based on appropriate schedules but few of them default their child from full vaccination without completing vaccines. … as we identified from integrated community-based supportive supervision through asking child mothers, some health workers are not properly telling the child mothers the type of vaccine received, not explaining the expected side effects of vaccines to the child mothers, and not properly telling child mothers about the next appointments are the main reasons for defaulting from full vaccination*.*”*

The 35-year-old health extension worker said that: “… *some of the child mothers have not vaccinated their children due to distance from health facility and inconvenient road conditions of the geographical area that causes defaulting of children from receiving all vaccines*.”

A 26-year-old EPI focal person mentioned that: “… *as to our catchment location we have a logistic problem and it is hard to reach area which is difficult to go by on foot walk to provide vaccines for children … and due to lack of motorcycle for supervision to ensure that all the children were vaccinated or not. There is also a lack of refreshment training on child vaccination for some health extension workers as a problem and I believe that these problems could be the barriers to childhood vaccination.*”

Another 32-year-old EPI focal person said that: “… *of our primary health care unit catchment area there is a shortage of human resources especially health extension workers at some health post only one health extension worker is working in a large population and densely populated kebele. Therefore, only one health extension worker per kebele cannot reach every child in such difficult conditions and this is a major cause of childhood vaccine defaulting and not full vaccination.*”

## Discussion

This study aimed to explore and describe barriers to full vaccination among children living in Siraro District, Ethiopia. This study identified the key barriers and challenges to the full vaccination status of the children. The identified barriers to full vaccination among children were related to the following five thematic areas: parent-related factors, knowledge and awareness, attitude and service satisfaction, rumors and misinformation, and vaccination service delivery-related factors.

The study revealed a strong interplay between the parent-related factors and the barriers to full vaccination. Despite this, the evidence from in-depth interviews of respondents indicated that family income, educational status, and geographical location of residence were the barriers to full vaccination. Some child caretakers are far away from the health facility and can afford to bring their children by transportation to vaccinate their children, but those who do not have the resources are less likely to take their children to the health facility.

Concerning this, the evidence from in-depth interviews of respondents suggested that the long walk to reach the health facility is a reason for child vaccination defaulting due to the distances from health institutions and inconvenient road conditions. Additionally, the mothers' understanding status and educational background are not similar in all groups, which may delay mothers from returning to the health facility for child vaccination (15, 23, 24).

In this study, the respondents mentioned that migration of parents from one place to other and from one region to other was found to be a barrier to full vaccination because some families live on the border area. The studies conducted in Sinana District (25), Somalia (24), and Afghanistan (26) supported this finding. This could be due to similar sociodemographic characteristics of the population that can lead to defaulting of the children from full vaccination.

The other ideas of respondents revealed that mothers/caretakers not having much understanding and knowledge of child vaccination was the key reason for not having full vaccination of children. This finding is consistent with the studies done in Kenya, Somalia, and Pakistan (24, 27, 28). This indicates that mothers who had insufficient knowledge of child immunization can lead to defaulting from the full vaccination of their children. This might be because some mothers who do not have more awareness and understanding of childhood vaccination and those who are not getting educated about immunization are not completing their child vaccination based on appropriate vaccine schedules. Those mothers who have knowledge and awareness of vaccination may be interested in vaccinating their children. Additionally, the other finding also supports these ideas, which indicates that when mothers do not understand and know more about child vaccination, they do not bring their children for vaccination, which is a reason for childhood vaccine defaulting (25).

The finding also showed that attitude and poor service satisfaction about the vaccination by mothers were the commonly reported beliefs among participants, which is consistent with the reports from other countries (14, 15, 27). This might be because those mothers who have a poor attitude and are not satisfied with the child vaccination service delivery are more likely to default their children from full vaccination.

This study also revealed that some mothers have misinformation and misunderstanding about child vaccination due to rumors from other neighbor parents, who say God will protect their child from illness; therefore, they believe that vaccination may cause physical disability in children. This finding is supported by studies done in Sinana District (25), Somalia (24), and Pakistan (27). This might be due to the parents not having enough understanding and awareness of vaccine-preventable diseases and the uses of child vaccination. Additionally, information dissemination on child vaccination by health workers may not reach the whole community to know more about the importance of childhood vaccination.

The study also identified that vaccination service delivery-related barriers to full vaccination were the other key reasons for preventing children from vaccination. The participants mentioned that closed health posts during visits to vaccinate the child, vaccine vials not opened for a few children, absence of health workers from health posts, fear of vaccine side effects, and vaccine stock out were the reported barriers to full vaccination. This finding is supported by the studies conducted in Sinana District (25), Arbegona District (20), Uganda (29), and Afghanistan (26).

This might be because the absence of health extension workers and closed health posts during repeated visits of mothers/caretakers for child vaccination may enhance the risk of defaulting from full vaccination. Additionally, they believe that the issue of vaccine vials not being opened for a few children and fear of side effects may not satisfy parents to complete child vaccination. This implies that the mothers/caretakers can default their children from completing full vaccination because they may not get appropriate information on vaccination about possible vaccine side effects and because health extension workers are not committed to providing immunization service at all times.

### Strengths and limitations of the study

Being a qualitative approach gives the strength to this study for better understanding and exploring barriers to full vaccination among children that by quantitative findings in previous studies cannot address. This study has its limitations of qualitative finding nature. Even though the study samples were determined by the idea saturation of the study participants, they might not be representative of the general and diverse population, and the results cannot be generalized to other settings. In addition, the study encountered limitations such as limited qualitative literature on vaccination to use in the Discussion section for a comparison of the finding with other articles. Instead, some mixed studies were used due to a limited number of qualitative articles on child vaccination.

## Conclusion

The evidence from the finding indicates that barriers to full childhood vaccination among children living in Siraro District were the reasons for defaulting of the children from vaccination. Child vaccination barriers such as forgetting the next vaccination schedule, migration of parents from one place to other, work overload, insufficient resources, lack of knowledge and awareness, poor attitude and satisfaction toward child vaccination, rumors and misinformation, inconvenient road conditions, and geographical location of residence. Additionally, vaccination service delivery-related barriers such as vaccine vials not being opened for a few children, fear of vaccine side effects, closed health posts during visits by child mothers for vaccination, and absence of health extension workers at health posts were the key barriers to childhood full vaccination.

The district health office should focus on strengthening health communication activities related to child vaccination by employing experts through local mass media to improve vaccination awareness and address concerns about vaccine side effects at the community level. Giving specific attention to the children found in hard-to-reach areas, focusing on the distribution of vaccination sites, and adding new vaccination sites as needed to address the issue of inconvenient road conditions and geographical areas will decrease barriers to childhood vaccination. Health professionals should work on transferring appropriate health information to the community about the nature of vaccines, the type of vaccine received, the next vaccination schedule, and the importance of childhood vaccination to address the issues of misinformation and rumors to prevent defaulting of the children from full vaccination. The Ethiopian Federal Ministry of Health and concerned stakeholders should work on substitution and availing of few-dose vials instead of the multidose vial to solve the issue of vaccine vials not being opened for a few children.

## Data Availability

The original contributions presented in the study are included in the article/Supplementary Material, further inquiries can be directed to the corresponding author.
